# Mouse Pharmacokinetics and In Vitro Metabolism of SH-11037 and SH-11008, Synthetic Homoisoflavonoids for Retinal Neovascularization

**DOI:** 10.3390/pharmaceutics14112270

**Published:** 2022-10-24

**Authors:** Eun-yeong Kim, Bit Lee, Sangil Kwon, Timothy W. Corson, Seung-Yong Seo, Kiho Lee

**Affiliations:** 1 College of Pharmacy, Korea University, Sejong 30019, Korea; 2 Institute of Pharmaceutical Science and Translational Research, Korea University, Sejong 30019, Korea; 3 College of Pharmacy, Gachon University, Incheon 21936, Korea; 4 Eugene and Marilyn Glick Eye Institute and Department of Ophthalmology, Indiana University School of Medicine, Indianapolis, IN 46202, USA; 5 Department of Biochemistry and Molecular Biology, Indiana University School of Medicine, Indianapolis, IN 46202, USA; 6 Department of Pharmacology and Toxicology, Indiana University School of Medicine, Indianapolis, IN 46202, USA; 7 Biomedical Research Center, Korea University Guro Hospital, Seoul 08308, Korea

**Keywords:** SH-11037, SH-11008, homoisoflavonoid, pharmacokinetics, drug metabolism, hydrolysis, carboxylesterase

## Abstract

Cremastranone is a member of the homoisoflavanone family with anti-angiogenic activity in the eyes. SH-11037, a potent and selective synthetic homoisoflavonoid derived from cremastranone, was studied here for pharmacokinetics and metabolism characterization with a special focus on esterase-mediated hydrolysis. SH-11037 was shown to be converted rapidly and nearly completely to SH-11008 following an intravenous dose in mice. SH-11008 showed a high systemic clearance well exceeding the hepatic blood flow in mice. Neither SH-11037 nor SH-11008 were detected in plasma following oral administration of SH-11037 and SH-11008 in mice. Carboxylesterase was shown to be responsible for the rapid and quantitative hydrolysis of SH-11037 to SH-11008 in mouse plasma; the hydrolytic bioconversion was much slower in dog and human plasma, with butyrylcholinesterase and paraoxonase 1 likely being responsible. In vitro metabolism studies with liver S9 fractions suggested that SH-11008 was likely to have a high hepatic metabolic clearance with a predicted hepatic extraction ratio close to 1 in both mouse and human. In conclusion, SH-11037 and SH-11008 both appear to possess pharmacokinetic profiles suboptimal as a systemic agent. SH-11008 is suggested to possess a low potential for systemic toxicity suitable as a topical ocular therapeutic agent.

## 1. Introduction

**Figure 1:**
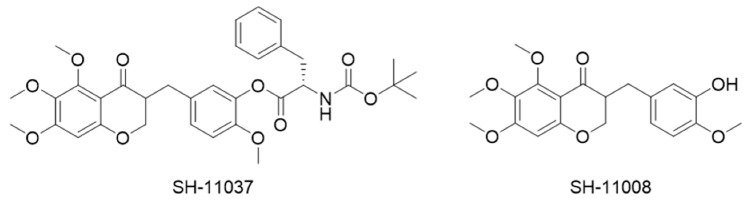
Chemical structures of SH-11037 and SH-11008.

Ocular neovascularization is associated with diseases such as wet age-related macular degeneration, retinopathy of prematurity, and proliferative diabetic retinopathy, leading to blindness in severe cases [1,2,3]. Cremastranone is a member of the homoisoflavanone family of natural products that have anti-angiogenic activity in the eyes [4,5,6,7]. We have reported previously the design, synthesis, and biological evaluation of novel homoisoflavonoid analogues using cremastranone as primary scaffold in an effort to discover a potent and selective inhibitor of angiogenesis [8]. Our structure–activity relationship analysis showed that the incorporation of N-carbamate amino acids improved the anti-angiogenic activity in vitro and in vivo [8]. Our study also showed that SH-11037 (Figure 1) was the most potent and selective inhibitor of neovascularization among the synthesized homoisoflavonoids [8,9].

We also have reported the results of our previous investigation on the pharmacokinetics and metabolism of cremastranone, showing that it had a high systemic clearance (CL) and a poor oral absorption, likely due to extensive metabolism in the liver and the intestine [10]. In this article, we report the pharmacokinetics and drug metabolism characteristics of SH-11037 with the aim to assist mainly in its evaluation as a systemic therapeutic agent. Mouse pharmacokinetics and various drug metabolism studies, including metabolic stability and metabolite identification, have been conducted similarly as reported previously with cremastranone [10]. In addition, we have conducted a thorough investigation on the possibility of hydrolytic metabolism as SH-11037 has hydrolyzable functional groups, namely an ester and a carbamate moiety (Figure 1), and many xenobiotics of similar characteristics are known to be hydrolyzed by various enzymes in the body [11,12,13]. We especially focused on esterases since they, as a major class of hydrolase expressed ubiquitously in various drug eliminating organs and tissues including the liver, kidney, intestine, and plasma, are known to be involved in the hydrolytic metabolism of a variety of xenobiotics [14,15]. The pharmacokinetics and metabolism properties of SH-11008 (Figure 1) and the hydrolytic metabolite of SH-11037, have also been characterized in this study as this compound has a potential for acting as an active metabolite in the body [8].

## 2. Materials and Methods

### 2.1. Materials

SH-11037 and SH-11008 were synthesized as reported previously [8]. The synthesis of SH-11008 was commenced with 5,6,7-trimethoxyphenol as a starting material through a 3-step sequence with acetylation, chromone formation using N,N-dimethylformamide dimethyl acetal, and hydrogenation to afford 5,6,7-trimethoxychroman-4-one. Aldol condensation of 5,6,7-trimethoxychroman-4-one with isovanillin and subsequent hydrogenation afforded SH-11008. With SH-11008 in hand, the esterification with Boc-L-Phe-OH afforded SH-11037. Glipizide, bis(4-nitrophenyl)phosphate (BNPP), 1,5-bis(4-allyldimethylammoniumphenyl) pentan-3-one (BW284c51), ethopropazine (EPZ), 5,5′-dithio-bis-2-nitrobenzoic acid (DTNB), uridine 5′-diphosphoglucuronic acid (UDPGA), β-nicotinamide adenine dinucleotide phosphate reduced (NADPH), 3′-phosphoadenosine-5′-phosphosulfate (PAPS), dimethylacetamide (DMA), and hydroxypropyl-β-cyclodextrin (HPβCD) were purchased from Sigma-Aldrich Korea (Yongin, Kyeonggi, Korea). Pooled male CD-1 mouse liver S9 (MLS9) fractions, pooled human liver S9 (HLS9) fractions and UDP-glucuronosyltransferase (UGT) Reaction Mix Solution B (250 mM Tris·HCl, 40 mM MgCl_2_, and 0.125 mg/mL alamethicin) were purchased from BD Korea (Seoul, Korea). Pooled mouse plasma was prepared in house using the blood obtained from male ICR mouse (8 weeks old). Pooled male beagle dog plasma (1.7~2.5 years old) was provided by KPC (Kyunggi, Korea). Pooled human plasma was purchased from Innovative Research (Novi, MI, USA). HPLC-grade water and acetonitrile (CH_3_CN) were from JT Baker (Phillipsburg, NJ, USA).

### 2.2. Mouse Pharmacokinetics

Pharmacokinetic studies were performed with male ICR mice (8 weeks old, 30~35 g) purchased from Koatech Co. (Kyunggi, Korea) as reported previously [10]. Test compounds were dissolved at 1 mg/mL in DMA/Tween 80/20% aqueous HPβCD (10/10/80 vol%) to prepare the dosing solutions. SH-11037 was administered by oral (p.o.) gavage at a dose of 10 mg/kg and a dose volume of 10 mL/kg (equimolar to 6 mg/kg of SH-11008) or intravenously (i.v.) via tail vein injection at a dose of 5 mg/kg and a dose volume of 5 mL/kg (equimolar to 3 mg/kg of SH-11008), respectively. SH-11008 was administered p.o. at a dose of 6 mg/kg and a dose volume of 6 mL/kg or i.v. at a dose of 3 mg/kg and a dose volume of 3 mL/kg, respectively. Plasma samples were analyzed as described below in Section 2.6.1.

### 2.3. Stability of SH-11037 in Phosphate Buffer and Plasma

SH-11037 (1 μM) was incubated in triplicate in 0.1 M phosphate buffer (pH 7.4) or in mouse, dog, and human plasma to investigate its stability. SH-11037 (40 μM) in 50 vol% aqueous CH_3_CN was mixed with 0.1 M phosphate buffer (pH 7.4) or plasma diluted 3-fold with 0.1 M phosphate buffer (pH 7.4) to a final volume of 160 μL and a final concentration of 1 μM. The resulting solutions were then incubated at 37 °C in a shaking water bath. The incubations were terminated at selected times by adding 160 µL of ice-cold CH_3_CN containing glipizide (500 ng/mL) as the internal standard (IS). The resulting mixture was vortexed, sonicated, and centrifuged at 3000× *g* for 20 min. Aliquots of the supernatant were subjected to LC-MS/MS analysis described below in Section 2.6.2.

### 2.4. Chemical Inhibition of SH-11037 Hydrolysis

Chemical inhibition assay was conducted in mouse, dog, and human plasma and mouse eye homogenate to identify the enzyme(s) involved in the hydrolysis of SH-11037. BW284c51 [16], DTNB [17], EPZ [18], and BNPP [19,20] were used as selective inhibitors of acetylcholinesterase (AChE), paraoxonase 1 (PON1), butyrylcholinesterase (BchE), and carboxylesterase (CES), respectively. Mouse eyes (male ICR mouse, 8 weeks old) taken immediately after CO_2_ asphyxiation were homogenized by an electric tissue homogenizer (Tissue Master-125, Omni International, Kennesaw, GA, USA) in an equal amount of 0.1 M potassium phosphate buffer (pH 7.4) on ice. The resulting homogenate was then centrifuged at 3000× *g* for 20 min. The supernatant was stored at −80 °C until the experiment. DMSO stock solutions of the inhibitors (0.4, 4 and 40 mM) were mixed with plasma diluted 3-fold with 0.1 M phosphate buffer (pH 7.4) to a final volume of 160 μL and final inhibitor concentrations of 0.01, 0.1, and 1 mM. The supernatant of mouse eye homogenate was treated similarly with only BNPP as the inhibitor and a single inhibitor concentration of 1 mM. The resulting pre-incubation solutions were then incubated for 2 h at 37 °C in a shaking water bath. SH-11037 (40 μM) in 50 vol% aqueous CH_3_CN was mixed with the pre-incubation solutions to a final volume of 160 μL and a final concentration of 1 μM. The resulting solutions were then incubated at 37 °C in a shaking water bath. The incubations were terminated at selected times by adding 160 µL of ice-cold CH_3_CN containing glipizide (500 ng/mL) as the IS. The resulting mixture was vortexed, sonicated, and centrifuged at 3000× *g* for 20 min. Aliquots of the supernatant were subjected to LC-MS/MS analysis described below in Section 2.6.2.

### 2.5. Metabolic Stability and Biotransformation of SH-11008

For studies using NADPH or PAPS as a cofactor, 4 μL of SH-11008 (40 μM) solution in 40 vol% CH_3_CN were mixed with 40 μL of 4 mg protein/mL HLS9 or MLS9 fractions diluted in 0.1 M potassium phosphate buffer (pH 7.4) and 100 μL of 0.1 M potassium phosphate buffer (pH 7.4). When using UDPGA as a cofactor, 32 μL of UGT Reaction Mix Solution B and 68 μL of de-ionized water were used instead of 100 μL of 0.1 M potassium phosphate buffer (pH 7.4). The mixture was warmed at 37 °C for 5 min. The reaction was then initiated by adding 16 μL of 10 mM cofactor dissolved in de-ionized water. The assay condition for UDPGA was used for metabolic stability studies conducted with a mixture of the three cofactors. The reaction was terminated at selected times by adding 160 μL of ice-cold CH_3_CN containing glipizide (500 ng/mL) as the IS. The resulting mixture was vortexed, sonicated, and centrifuged at 3000× *g* for 20 min. Aliquots of the supernatant were subjected to LC-MS/MS analysis described below in Section 2.6.2.

### 2.6. Sample Analysis

#### 2.6.1. Mouse Pharmacokinetics

Calibration standards and quality control (QC) samples were prepared by spiking 15 μL of blank plasma with 5 μL of analyte working solution prepared in 50 vol% aqueous CH_3_CN. For sample preparation, 15 μL aliquots of plasma samples combined with 5 μL of 50 vol% aqueous CH_3_CN were mixed with 3 volumes of ice-cold CH_3_CN containing glipizide (500 ng/mL) as the IS. The mixture was vortexed, sonicated, and centrifuged at 3000× *g* for 20 min. The supernatant was then mixed with the same volume of HPLC grade water before analysis. Sample analyses were conducted using Agilent 1290 Infinity HPLC system coupled to Agilent 6460 Triple Quadrupole LC-MS/MS system equipped with Jet Stream ESI ion source (Agilent Technologies Korea, Seoul, Korea). Chromatographic separation was performed on an Agilent Eclipse Plus C18 column (2.1 × 100 mm, 3.5 μm) with a Phenomenex Security Guard C18 guard column (4 × 20 mm) maintained at 40 °C. The mobile phase consisted of 0.1% formic acid in deionized water (A) and 0.1% formic acid in CH_3_CN (B): 0~2 min 5% B, 2~3 min to 95% B, and 3~6.5 min 95% B. Total run time including a 3 min equilibration time was 9.5 min. The flow rate was 0.45 mL/min. The injection volume was 5 µL. Detection of the analyte ions was performed in the positive multiple reaction monitoring (MRM) mode, with the following mass transitions (precursor > product): m/z 644 > 588, 397 > 367, and 446 > 321 for SH-11037, SH-11008 and IS, respectively. The developed analytical method was specific as no interfering peaks were observed in the chromatograms of the blank samples. The lower limit of quantitation of SH-11037 and SH-11008 were 0.24 and 0.48 ng/mL, respectively, with a signal-to-noise ratio based on root mean square noise > 10. The calibration curves were linear in the range of 0.24–500 ng/mL (r^2^ > 0.997) for SH-11037 and 0.48–500 ng/mL (r^2^ > 0.998) for SH-11008, respectively. The precision and accuracy of the analytical method were evaluated with the QC samples prepared in triplicate at 5, 250, and 400 ng/mL for both of SH-11037 and SH-11008: the coefficient of variation (%CV) and the relative error (%RE) were in the ranges of −21.23~5.72% and 1.47~12.30% for SH-11037 and −22.85~4.96% and 1.2~13.8% for SH-11008, respectively. Extraction recovery, matrix effect, and process efficiency were in the ranges of 91.8~107.1%, 86.3~99.9%, and 88.8~100.8% for SH-11037 and 88.9~103.8%, 88.9~100.0%, and 80.0~97.4% for SH-11008, respectively. Quantitative analysis data were obtained with Agilent Mass Hunter Quantitative Analysis QQQ (ver. B.05.00) software.

#### 2.6.2. In Vitro Studies

The LC-MS/MS system consisted of Agilent 1290 Infinity HPLC system and Agilent 6530 Accurate-Mass Quadrupole Time-of-Flight (Q-TOF) mass spectrometer equipped with dual AJS ESI ion source (Agilent Technologies Korea, Seoul, Korea). Chromatographic separation was performed on an Agilent Eclipse Plus C18 column (2.1 × 100 mm, 3.5 μm) with a Phenomenex Security Guard C18 guard column (4 × 20 mm) maintained at 40 °C. The mobile phase consisted of 0.1% formic acid in deionized water (A) and 0.1% formic acid in CH_3_CN (B): 0~2 min 5% B, 2~7 min to 95% B, and 7~10 min 95% B. Total run time including a 5 min equilibration time was 15 min. The flow rate was 0.4 mL/min. The injection volume was 5 µL. The mass spectrometer was operated in a positive Auto MS/MS scan mode with a full scan mass range of m/z 100–1000, fragmentor voltage 150 V, and CE 30 eV, selecting the two most intense precursor ions for collision-induced dissociation. Putative metabolites were identified using Agilent Mass Hunter Metabolite ID software (ver.B.04.00) followed by manual interpretation of the spectral data. Chromatograms and mass spectra were extracted using Agilent Mass Hunter Qualitative Analysis software (ver. B.05.00).

### 2.7. Data Analysis

Pharmacokinetic parameters were calculated by noncompartmental analysis of plasma concentration versus time profiles using PKSolver [21]. The scaling factors of mice and human, half-life (t_1/2_), intrinsic clearance (CL_int_), hepatic clearance (CL_H_), and hepatic extraction ratio (E_H_) were from the in vitro studies as described previously [10]. The fraction of dose hydrolyzed to and systemically available as a metabolite was demonstrated previously [22,23]. The fraction of parent drug converted to the metabolite (fm) was calculated as follows:

fm (%) = (AUC(m) × CL(m))/D × MW_parent_/MW_metabolite_ × 100, (1)

where D is dose of the parent drug, AUC(m) is area under the curve for the metabolite after a dose of parent drug, CL(m) is CL of metabolite, and MW is molecular weight. 

## 3. Results

### 3.1. Pharmacokinetics of SH-11037 and SH-11008 in Mice

The pharmacokinetic characteristics of SH-11037 and SH-11008 were investigated in mice following a single i.v. and p.o. dose. 

**Figure 2:**
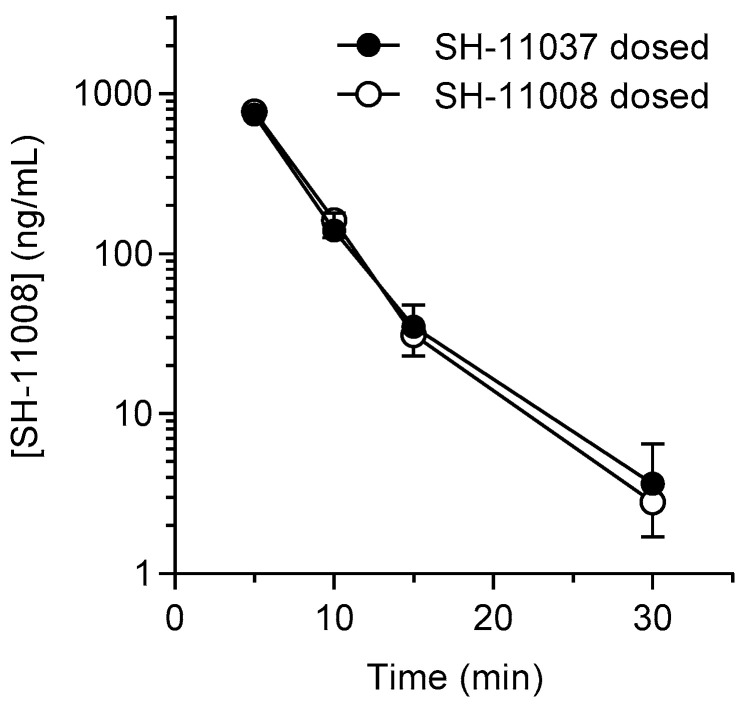
Plasma concentration–time curves of SH-11008 following a single i.v. dose of SH-11037 and SH-11008 in mice. Plasma samples were collected for 24 h following a single i.v. dose of SH-11037 (5 mg/kg) and SH-11008 (3 mg/kg, equimolar to 5 mg/kg SH-11037) in male ICR mice. SH-11037 was BQL throughout the time course. SH-11008 was BQL after 30 min post-dosing. Data are presented as mean ± standard deviation (S.D.) (n = 3).

The plasma levels of SH-11037 were below the quantitation limit (BQL, <0.24 ng/mL) throughout the 24-h time course following a single i.v. dose (5 mg/kg) in mice; SH-11008 was detected above its quantitation limit in the plasma up to 30 min post-dosing with the peak time (t_max_) being the first time point (5 min) (Figure 2). The plasma concentration–time curve obtained following a single i.v. dose of SH-11008 (3 mg/kg) was almost identical with the curve from the equimolar dose of SH-11037 (5 mg/kg) (Figure 2), indicating that SH-11037 was converted rapidly and nearly completely to SH-11008 in the body. In fact, the calculated fm of the parent drug (SH-11037) converted to the metabolite (SH-11008) was close to 100% (Table 1). SH-11008 showed a high CL of 210.0 ± 17.2 mL/min/kg, well exceeding the hepatic blood flow (~90 mL/min/kg). 

SH-11037 was BQL in the plasma throughout the time course following a single p.o. dose (10 mg/kg). The plasma levels of SH-11008 were also BQL (<0.48 ng/mL) following a single p.o. dose of SH-11037 (10 mg/kg) and SH-11008 (6 mg/kg, equimolar to 10 mg/kg SH-11037). Pharmacokinetic parameters of SH-11037 and SH-11008, therefore, could not be determined after the p.o. doses.

### 3.2. Hydrolysis of SH-11037 in Mouse, Dog, and Human Plasma

**Figure 3:**
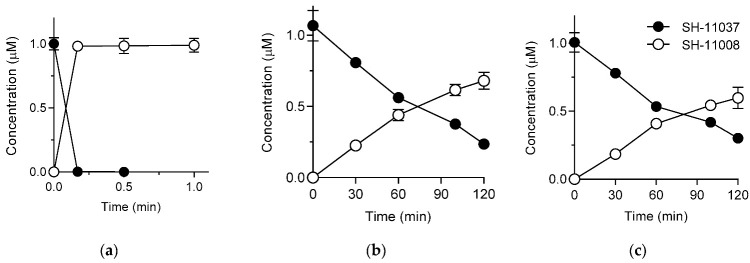
Stability of SH-11037 in (**a**) mouse, (**b**) dog, and (**c**) human plasma. SH-11037 (1 µM) was incubated in triplicate in plasma at 37 °C. SH-11037 and SH-11008 were quantitated simultaneously at selected times during the incubations. Data are mean ± S.D. (n = 3).

The differences in the stability of SH-11037 in enzymatic and non-enzymatic environments were examined by a comparison of their hydrolysis in potassium phosphate buffer (pH 7.4), as well as in mouse, dog, and human plasma. SH-11037 was stable in the pH 7.4 buffer with nearly 100% remaining after 1 h incubation at 37 °C; correspondingly, SH-11008 was not detected following the same incubation. On the contrary, SH-11037 was hydrolyzed quantitatively to SH-11008 in mouse, dog, and human plasma with a half-life (t_1/2_) of 0.018, 69.1, and 73.2 min, respectively (Figure 3). These results suggested that SH-11037 was hydrolyzed enzymatically to SH-11008 in the plasma.

### 3.3. Effect of Esterase Inhibitors on SH-11037 Hydrolysis

Chemical inhibition studies were conducted using various esterase inhibitors in an effort to identify the enzyme(s) involved in the hydrolysis of SH-11037 in the plasma, especially in its rapid hydrolysis in mouse plasma, and to understand the reasons for the significant species difference observed between the rodent and nonrodents.

The CES inhibitor, BNPP, showed a statistically significant inhibition of the conversion of SH-11037 to SH-11008 in mouse plasma in a concentration-dependent manner (Table 2); BNPP completely inhibited the SH-11008 formation at 1 mM suggesting that the hydrolysis was mediated predominantly by CES. BNPP, on the other hand, caused no significant inhibition in SH-11037 hydrolysis in dog and human plasma (Table 2), consistent with the low plasma levels of CES reported in these animal species [15,24]. SH-11037 hydrolysis, instead, was inhibited by the PON1 inhibitor, DTNB, and the BChE inhibitor, EPZ, in dog plasma (Table 2). In human plasma, only DTNB showed a concentration-dependent inhibition in the hydrolysis (Table 2). The AChE inhibitor, BW284c51, did not inhibit the hydrolysis in any of the species tested in this study (Table 2). Taken together, CES appears to be responsible for both the rapid hydrolysis in mouse plasma and the species difference between the rodent and nonrodents.

**Figure 4:**
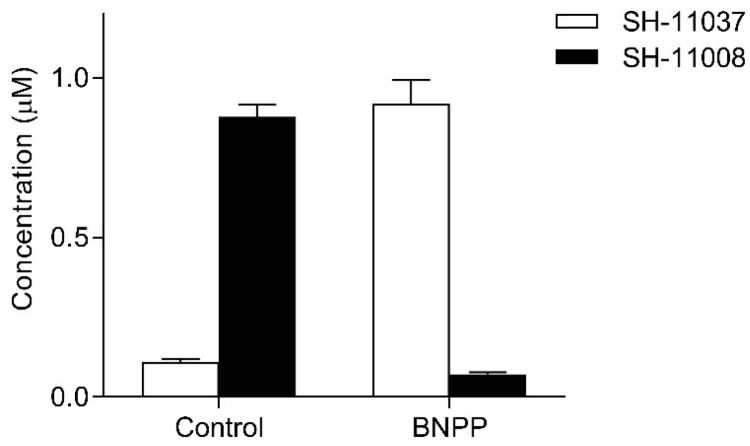
Effect of esterase inhibitors on the stability of SH-11037 in mouse eye homogenate. SH-11037 (1 μM) was incubated in triplicate in pooled mouse eye homogenate for 1 min at 37 °C following 2 h preincubation in the absence (control) and presence of BNPP (1 mM). Data are mean ± S.D. (n = 3).

Since SH-11037 may be applied directly to eyes as an ophthalmic drug candidate, we also examined its stability in mouse eye homogenate. SH-11037 was hydrolyzed rapidly also in mouse eye homogenate with ~90% being converted to SH-11008 within 1 min (Figure 4). BNPP inhibited the hydrolysis nearly completely during the incubation, indicating that CES was again the predominant enzyme responsible for the hydrolysis in the eyes (Figure 4).

### 3.4. Metabolic Stability of SH-11008

**Figure 5:**
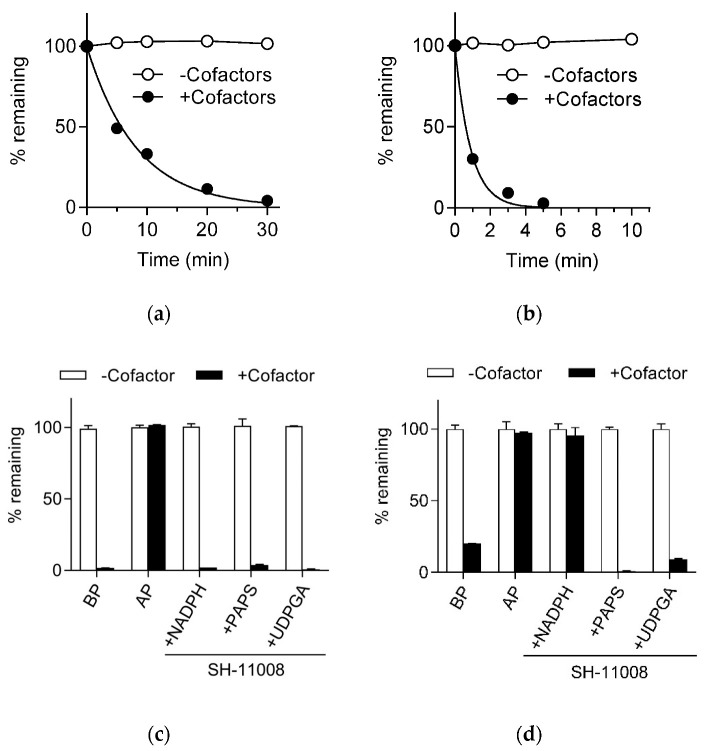
Metabolic stability of SH-11008 in MLS9 (**a**,**c**) and HLS9 (**b**,**d**) fractions. SH-11008 (1 µM) was incubated in triplicates at 37 °C in the liver S9 fractions (1 mg protein/mL) in the absence and presence of the cofactors (NADPH, PAPS, and UDPGA). The cofactors were added at 1 mM either as a mixture (**a**,**b**) or individually (**c**,**d**). Buspirone (BP) and antipyrine (AP) incubated in the presence of NADPH (**c**,**d**) were used as positive and negative control, respectively. The incubations in (**c**,**d**) were conducted for 30 min. Data are mean ± SD (n = 3).

The metabolic stability of SH-11008 was studied in vitro using MLS9 and HLS9 fractions fortified with a mixture of NADPH, UDPGA, and PAPS as cofactors for cytochrome P450 (CYP), UDP-glucuronosyltransferase (UGT), and sulfotransferase (SULT), respectively (Figure 5a,b). SH-11008 was metabolized rapidly with a t1/2 of 5.7 and 0.6 min after incubation in MLS9 and HLS9 fractions, respectively (Figure 5a,b; Table 3). As a result, SH-11008 showed a high predicted CL_H_, nearly identical to the hepatic blood flow in both mice and humans. Correspondingly, its E_H_ values were close to 1 (Table 3). The observed discrepancies in CL_int_ and CL_H_ between mice and humans (Table 3) were due to differences in the scaling factors used in the calculations (see Section 2.7).

SH-11008 was also incubated in the same manner with a cofactor added individually to identify the metabolic enzyme(s) involved. As shown in Figure 5c, SH-11008 was metabolized rapidly in the presence of NADPH, UDPGA, or PAPS in MLS9 fractions, suggesting that CYP450, UGT, and SULT were involved in its biotransformation in mouse liver. Interestingly, only the phase II biotransformation reactions (i.e., glucuronidation and sulfation) appeared to be responsible for the rapid and extensive metabolism of SH-11008 in HLS9 fractions as it was shown to be stable in the presence of NADPH (Figure 5d).

### 3.5. Identification of SH-11008 Metabolites

**Figure 6:**
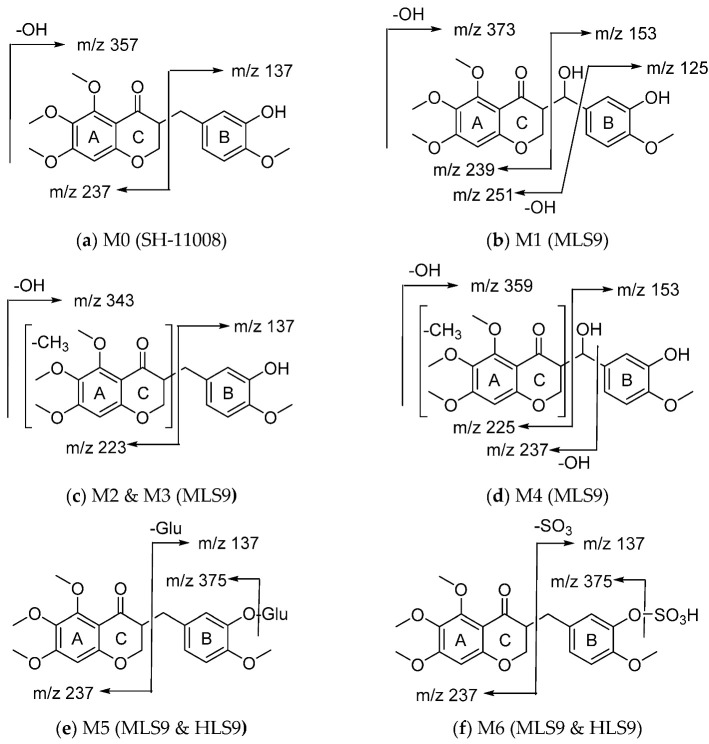
Proposed structures and mass fragmentation patterns of putative SH-11008 metabolites identified using MLS9 and HLS9 fractions. SH-11008 metabolites were identified as described in Table 4. Glu: glucuronic acid.

Metabolites of SH-11008 were identified tentatively by a Q-TOF LC-MS/MS system following the incubations described in Figure 5 (see Section 2.6.2 for experimental details). A total of four phase I (M1–M4) and two phase II metabolites (M5 and M6) of SH-11008 were identified in this study (Table 4; Figure 6). None of these putative metabolites were detected in the control samples which lacked the cofactors, indicating that they were generated by enzyme-mediated biotransformation reactions.

The [M + H]^+^ ion of SH-11008 was detected at m/z 375.1426 (Table 4). SH-11008 produced a fragment ion of m/z 357 after the loss of OH, and C-C bond cleavage between the B- and C-rings generated the fragment ions of m/z 237 and 137 (Figure 6a). The phase I metabolites (M1–M4) were detected in the MLS9 fractions following the incubation of SH-11008 in the presence of NADPH, but not in the HLS9 fractions (Figure 6b–d), consistent with the metabolic stability results described in Figure 5. The [M + H]^+^ ion of M1 was detected at m/z 391.1363 corresponding to a mono-oxygenated metabolite (i.e., +16 u from the parent) of SH-11008 (Table 4). M1 produced a fragment ion of m/z 373 after the loss of OH (Figure 6b). The fragment ions of M1 at m/z 153 (16 u higher than the fragment ion m/z 137 of SH-11008) and m/z 125 suggest that the oxygenation likely occurred on the carbon between the B- and C-rings (Figure 6b). The [M + H]^+^ ions were detected at m/z 361.1232 for M2 and at m/z 361.1290 for M3, corresponding to the mono-demethylated metabolites (i.e., −14 u from the parent) of SH-11008 (Table 4). M2 and M3 produced a fragment ion of m/z 343 after the loss of OH, and C-C bond cleavage between the B- and C-rings generated the fragment ions of m/z 223 and 137 (Figure 6c). The fragment ion at m/z 223 of M2 and M3, which was 14 u lower than the one at m/z 237 of SH-11008, indicates that the A-ring of M2 and M3 underwent loss of a methyl group (Figure 6c). M2 and M3 had the same mass but different retention times (Table 4). Unfortunately, since both metabolites share the same fragment ions, the exact metabolic position could not be specified (Table 4). The [M + H]^+^ ion of M4 was detected at m/z 377.1218 corresponding to a concurrent metabolite of mono-oxygenation and mono-demethylation (i.e., +2 u from the parent) of SH-11008 (Table 4). M4 produced a fragment ion of m/z 359 after the loss of OH (Figure 6d). M4 also had fragment ions at m/z 225 and 237, which was 14 u lower than the fragment ions of M1 observed at m/z 239 and 251, consistent with mono-demethylation (Figure 6d). The fragment ions at m/z 153 and 237 suggest that the mono-oxygenation of M4 occurred on the same position as M1 (Figure 6d). M5 and M6 were detected in both MLS9 and HLS9 fractions. The [M + H]^+^ ions were detected at m/z 551.1758 for M5 and at m/z 455.1015 for M6 corresponding to a mono-glucuronide conjugate (i.e., +176 u from the parent) and a mono-sulfate conjugate (i.e., +80 u from the parent) (Table 4). Fragmentation of M5 and M6 generated the [M + H]^+^ ion of SH-11008 (m/z 375) together with its fragment ions (m/z 237 and 137) (Figure 6e,f).

**Scheme 1:**
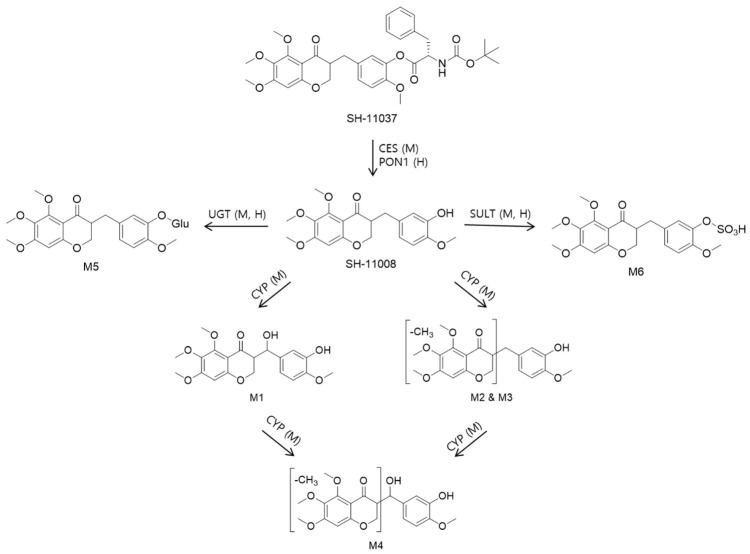
Proposed metabolic pathway of SH-11037. M: mouse, H: human, Glu: glucuronic acid.

A proposed biotransformation pathway of SH-11037 based on the current results is shown in Scheme 1.

## 4. Discussion

SH-11037 has been shown to be converted rapidly in a quantitative manner to SH-11008 in mice following an i.v. dose in this study. The in vitro stability data showing a rapid and quantitative hydrolysis of SH-11037 to SH-11008 in mouse plasma suggested that the hydrolysis likely occurred mainly in the plasma following the i.v. administration. The in vitro chemical inhibition studies demonstrated that CES was responsible for the hydrolysis of SH-11037 in mouse plasma; this esterase also seems to have caused the species difference in plasma stability between the rodent and nonrodents, as previous reports have shown that it is highly expressed in mouse plasma, but not in dog and human plasma [15,24].

SH-11008 was reported previously to show a good inhibitory activity on human microvascular retinal endothelial cell proliferation; although less potent than SH-11037 [8]. As this result suggests that SH-11008 may work as an active metabolite of SH-11037 in the body, we characterized its metabolism and pharmacokinetic properties in this study. SH-11008 had a high CL in mice consistent with its rapid metabolism in MLS9 fractions predictive of a high CL_H_. Neither SH-11037 nor SH-11008 were detected in plasma following a p.o. dose of SH-11037 in mice, presumably due to extensive first-pass metabolism of both the parent and the metabolite in the gut, blood vessels, and the liver; this result again is consistent with the in vitro metabolic stability results.

It can be said that SH-11037 is not suitable as a therapeutic agent for systemic delivery based on the current metabolism and pharmacokinetics results obtained in mice. However, the in vitro plasma stability results provide the possibility that its pharmacokinetics in nonrodents (especially in human) may be significantly different from the mice, i.e., it may have a significantly lower CL and a higher oral bioavailability in nonrodents leading to a higher and sustained plasma exposure sufficient for a systemic agent. In addition, caution needs to be taken when mouse and other rodents are used in preclinical pharmacokinetics, pharmacodynamics, and toxicity/safety studies as these animal models may lack clinical relevance due to significant species difference in pharmacokinetics.

The current study also showed that SH-11037 was rapidly hydrolyzed to SH-11008 in mouse eye homogenate, again most likely by CES. Although this result suggests the possibility that SH-11037 can serve as a prodrug of SH-11008 for ocular delivery, such as eye drop and intravitreal injection, a potential significant species difference in ocular CES activity needs to be addressed thoroughly prior to further investigation.

SH-11008 was shown to be cleared highly efficiently in mice following a systemic administration as described earlier, and our in vitro studies have demonstrated that rapid metabolism was likely responsible for the high in vivo CL. SH-11008 also has been predicted to have a high CL in human plasma from the in vitro metabolic stability results. Additionally, it was not detected in plasma following a p.o. administration in mice. Taken together, it would be inappropriate to move forward with this compound as a drug candidate for a systemic therapeutic agent. On the other hand, SH-11008 may serve as a promising lead candidate for ocular delivery as its extensive hepatic metabolism is likely to keep the systemic exposure minimal; therefore, minimizing the possibility of systemic toxicity.

Metabolite identification was conducted for SH-11008 as SH-11037 was converted rapidly and quantitatively to SH-11008. It is interesting to note that CYP was involved in the metabolism of SH-11008 in mouse plasma, but not in human plasma. With the exact reason underlying this species difference remaining to be elucidated, it will be necessary to take caution in extrapolating any mouse data for SH-11008 into human data as its disproportionate metabolites formed only in mouse plasma may complicate the interspecies correlations. Our in vitro results have shown that SH-11008 was metabolized rapidly in HLS9 fractions due predominantly to phase II biotransformation reactions on the phenol group of the C ring. Therefore, it would be plausible to modify the metabolic ‘soft spot’ to derive a drug candidate with an optimal metabolic stability suitable for systemic delivery.

In conclusion, the metabolism and mouse pharmacokinetics of SH-11037, a synthetic homoisoflavonoid, were characterized in this study to support its evaluation as a potential therapeutic agent for retinal neovascularization. SH-11037 and its hydrolytic metabolite, SH-11008, both appear to possess pharmacokinetic profiles suboptimal as a systemic agent. SH-11037 is suggested, based on the current results, to possess a low potential for systemic toxicity and, therefore, considered to be suitable as a topical ocular therapeutic agent. The identified metabolites of SH-11008 provide metabolic ‘soft spot’ information that could be utilized for further medicinal chemistry efforts to derive a drug candidate for systemic delivery. Species differences in the biotransformation of SH-11037 and SH-11008 characterized in this study will need to be considered in their future evaluations as potential therapeutic agents.

## Figures and Tables

**Table 1:** Pharmacokinetic parameters of SH-11008 following a single i.v. dose of SH-11037 and SH-11008 in mice.

Parameter	SH-11037 Dosed	SH-11008 Dosed
Dose (mg/kg)	5	3
t_1/2_ (min)	3.6 ± 0.3	3.5 ± 0.5
t_max_ (min)	5.0 ± 0.0	5.0 ± 0.0
C_max_ (ng/mL)	738.1 ± 127.1	769.8 ± 1163.6
AUC_last_ (ng·min/mL)	14,709.3 ± 1855.8	14,333.8 ± 30.4
AUC_inf_ (ng·min/mL)	14,728.5 ± 1870.9	14,348.3 ± 1157.2
CL (mL/min/kg)	NA	210.0 ± 17.2
V_ss_ (L/kg)	NA	0.5 ± 0.1
fm (%)	102.7 ± 13.0	NA

Pharmacokinetic parameters were calculated by noncompartmental analysis of the plasma concentration–time curves presented in Figure 2. Values are mean ± S.D. (n = 3). NA: not applicable.

**Table 2:** Effect of esterase inhibitors on the hydrolysis of SH-11037 in mouse, dog, and human plasma.

Inhibitor (mM)	SH-11008 Formation (%) ^a^		
	Mouse	Dog	Human
Control (none)	98.3 ± 2.8	91.7 ± 1.2	91.7 ± 1.2
BW284c51 (0.01)	100.5 ± 5.5	102.0 ± 6.2	88.3 ± 1.2
BW284c51 (0.1)	101.9 ± 6.6	101.7 ± 2.3	87.3 ± 2.1
BW284c51 (1)	101.1 ± 2.0	89.0 ± 6.6	86.3 ± 6.4
DTNB (0.01)	102.1 ± 3.7	84.3 ± 2.3	84.3 ± 1.5
DTNB (0.1)	100.0 ± 0.1	62.7 ± 6.4	61.7 ± 6.7
DTNB (1)	96.2 ± 2.1	35.0 ± 6.2	12.0 ± 2.6
EPZ (0.01)	96.3 ± 1.3	99.0 ± 10.4	80.3 ± 5.5
EPZ (0.1)	95.7 ± 7.1	86.0 ± 5.3	97.0 ± 1.0
EPZ (1)	92.6 ± 4.6	51.7 ± 1.2	71.0 ± 3.5
BNPP (0.01)	98.5 ± 3.6	93.0 ± 12.3	92.0 ± 5.0
BNPP (0.1)	76.4 ± 3.6	83.3 ± 0.6	88.7 ± 2.1
BNPP (1)	ND	88.7 ± 11.0	85.3 ± 4.0

SH-11037 (1 µM) was incubated in triplicate in pooled mouse, dog, and human plasma for 1 min (mouse) and 2 h (dog and human) at 37 °C in the absence (control) and presence of esterase inhibitors. Data are mean ± S.D. (n = 3). ^a^ % of SH-11037 converted to SH-11008 during the incubations. ND: not detected. Data are mean ± S.D. (n = 3).

**Table 3:** Prediction of in vivo CL_H_ from in vitro metabolic stability for SH-11008.

Species	t_1/2_	CL_int_	CL_H_	E_H_
	(min)	(mL/min/kg)	(mL/min/kg)	
Mouse	5.7	1510.3	84.9	0.94
Human	0.6	3577.0	19.9	0.99

t_1/2_ was determined in vitro in MLS9 and HLS9 fractions as described in Figure 5. CL_H_ was estimated using the ‘well-stirred’ model of the liver (see Section 2.7).

**Table 4:** Mass spectral data of putative SH-11008 metabolites identified using MLS9 and HLS9 fractions.

No.	Biotransformation	Formula	[M + H]^+^^a^ (m/z)	△m ^b^ (ppm)	t_r_^c^ (min)	Product Ions (m/z)
M0	Parent	C_20_H_22_O_7_	375.1426	−3.4	6.70	357, 237, **137**
M1	Oxygenation	C_20_H_22_O_8_	391.1363	−6.3	6.15	373, **251**, 239, 153, 125
M2	Demethylation	C_19_H_20_O_7_	361.1232	−13.8	6.13	343, 223, **137**
M3	Demethylation	C_19_H_20_O_7_	361.1290	2.3	6.20	343, 223, **137**
M4	Oxygenation + demethylation	C_19_H_20_O_8_	377.1218	−3.4	5.66	359, 237, **225**, 153
M5	Glucuronidation	C_26_H_30_O_13_	551.1758	−0.1	5.80	**375**, 237, 137
M6	Sulfation	C_20_H_22_O_10_S	455.1015	1.8	6.37	375, 237, **137**

SH-11008 (1 µM) was incubated with MLS9 or HLS9 fractions (1 mg protein/mL) for 30 min at 37 °C in the presence of 1 mM NADPH, UDPGA, and PAPS as cofactors. Putative metabolites were identified by a Q-TOF LC-MS/MS system run in a positive Auto MS/MS mode. ^a^ Observed mass. ^b^ Deviation of the observed mass from the theoretical mass. ^c^ Retention time on chromatograms. Bold: most abundant product ions.

## Data Availability

Not applicable.
